# Psychosocial barriers and facilitators for a successful return to work following injury within firefighters

**DOI:** 10.1007/s00420-021-01712-z

**Published:** 2021-05-11

**Authors:** Liam Noll, Adrian Mallows, Jason Moran

**Affiliations:** School of Sport, Rehabilitation and Exercise Sciences, University of Essex, Colchester, Essex, CO4 3SQ UK

**Keywords:** Firefighter, Return from injury, Semi-structure interview, United Kingdom

## Abstract

**Objective:**

The aim was to explore firefighter’s experiences during their recovery from injury. Focused specifically on exploring perceived psychosocial barriers and facilitators firefighters faced during recovery and return to work.

**Methods:**

Semi-structured interviews were used to provide an in-depth understanding of the firefighter’s experiences. The semi-structured interviews were informed by a topic guide. The topic guide focused on five main themes, (1) overall experience of returning to operational duties following an injury, (2) perceived barriers experienced during their return to work, (3) perceived facilitators experienced during their return to work, (4) confidence in participating in physical activity following injury and (5) where they felt areas of improvement could be made with the return to work process. Thematic analysis of the data collected was undertaken using The Framework Method.

**Results:**

Two main themes were sought after transcription: barriers and facilitators. From these, nine subthemes were identified (1) communication, (2) confidence in physical activity participation, (3) modified duties, (4) physiotherapy, (5) return to operational duties, (6) support, (7) inconsistency, (8) use of station gyms, (9) detachment from the watch.

**Conclusions:**

Consideration should be made for the consistency of procedures followed during an individual’s return to work following an injury. Further research is needed to understand if the themes identified in this study are the same for other fire services. Further research is also needed to understand how the findings may be best implemented within the fire service.

**Supplementary Information:**

The online version contains supplementary material available at 10.1007/s00420-021-01712-z.

## Introduction

The recovery from injury and return to work is complex (Cancelliere et al. 2016). For firefighters, the physical demands of the job and the need for recovery to meet these demands is well documented (Stevenson et al. 2016; Smith 2011). Government statistics showed that 2466 firefighters in the United Kingdom suffered an injury between 2019–20 (Home Office 2020). Return to work for firefighters following common occupational-related injuries, such as musculoskeletal strains and sprains and stress fractures (Orr et al. 2019), can take from 3 to 12 weeks (Wright-Carpenter et al. 2004; Matheson et al. 1987). Re-injury rates for musculoskeletal sprains and strains are reported between 7 and 34% (Schmitt et al. 2012; Holme et al. 1999) and stress fractures have been reported at 29% (Ekstrand and Torstveit 2012). Such high re-injury rates suggest that current processes are suboptimal and the need to understand factors which influence a successful return work. A recent consensus study highlighted the need for a physical return to work assessment for firefighters following an injury, assessing physical parameters including muscular strength and aerobic fitness (Noll et al. 2021). Physical assessments including hose running, hose carrying, ladder lifting, ladder climbing and casualty evacuation were agreed to be included during a firefighters return to work process (Noll et al. 2021). Other factors including social support (Prang et al. 2015), and psychological factors including fear of re-injury and stress (Hsu et al. 2017) also need to be considered.

Negative psychological responses can lead to low levels of self-esteem as well as feelings of anxiety, depression and increased stress (Smith 1996). Progression through rehabilitation and recovery can be negatively affected by increased stress levels (Crossman 1997). Negative responses have been shown to peak at two particular points (Morrey et al. 1999); when the injury occurred and when the individual is allowed to return to physical activity in the same capacity before becoming injured (Hsu et al. 2017).

Fear of re-injury is an example of a negative response which can be a common factor amongst individuals returning to physical activity (Hsu et al. 2017). Despite pain resolving and function and strength returning, hesitancy to return to physical activity due to a fear of re-injury can remain (Schilaty et al. 2016; Russell et al. 2018). Reasons can include increased anxiety and catastrophic thinking (Fischerauer et al. 2018) which can decrease motivation to return to physical activity (Barber-Westin et al. 2019). In addition, previous experience of injury has been documented to relate to a feeling of ‘coming to terms’ with the injury and reduce motivation to meet the demands required to return to pre-injury status (Podlog and Eklund 2005). This decrease in motivation can then lead to physical inactivity (Barber-Westin et al. 2019).

Physical inactivity decreases aerobic fitness and strength levels (Kulinski et al. 2014; Leblanc et al. 2015). Decreased fitness and strength levels negatively impacts on firefighters performance level and safety when completing job-related tasks (Smith 2011). Included tasks involve hose running, hose carrying, ladder lifting, ladder climbing and casualty evacuation (Stevenson et al. 2016). The majority of operational tasks are completed by a firefighter within a group setting with other firefighters on duty with them (Podlog and Eklund 2005). The duty system is also known as a watch and firefighters can spend a long time working with the same watch, attending to both physically and psychologically challenging incidents (Johnston and Carroll 1998). This contributes to creating strong bonds and friendships between them (Johnston and Carroll 1998).

A reduction in social contact with colleagues whilst being off work injured can cause feelings of frustration due to the sudden lack of involvement (Sonnentag and Fritz 2007). Being away from colleagues due to injury can create a feeling of psychological detachment, which can be related to a reduced sense of wellbeing (Bianco 2001). Social support during recovery from an injury can increase motivation and a sense of inclusion, in addition to decreasing symptoms of depression and anxiety when returning to physical activity (Yang et al. 2014; Carless et al. 2013; Gill et al. 2008).

There is limited research focused on firefighters in the United Kingdom returning from work following an injury. The importance of understanding psychological and social factors for a successful return to work is clear from other active populations such as athletes and military personnel (Hsu et al. 2017; Smith 1996; Crossman 1997; Morrey et al. 1999; Schilaty et al. 2016; Russell et al. 2018; Fischerauer et al. 2018; Barber-Westin et al. 2019; Podlog and Eklund 2005; Yang et al. 2014), but to date this has not been investigated with firefighters. The aim of this study was to explore firefighter’s experiences during recovery from injury. Specifically, we sought to explore perceived psychosocial barriers and facilitators firefighters faced during recovery and return to work.

### Ethical approval

Ethical approval was sought and granted on 26th June 2020 by the University of Essex research ethics committee. Ethics reference; ETH1920-1683.

## Methods

### Study design

This study used semi-structured interviews to provide an in-depth understanding. A post-positivist perspective was used to underpin the design of this project.

The study is reported in accordance with the consolidated criteria for reporting qualitative (COREQ) research guidance (Tong et al. 2007).

### Data collection

Semi-structured interviews were informed by a topic guide (O'keeffe et al. 2015). The topic guide was developed by the chief investigator (LN) and was focused on five themes for a firefighter returning to operational duties following an injury: (1) overall experience of returning to operational duties following an injury, (2) perceived barriers experienced during their return to work, (3) perceived facilitators experienced during their return to work, (4) confidence in participating in physical activity following injury and (5) where they felt areas of improvement could be made with the return to work process [Appendix 1].

The interviews were conducted one to one with LN as the interviewer. LN is a male PhD research candidate who had received training in conducting semi-structured interviews. Both LN and the participants in this study were employed by the fire service, LN was a member of the support staff team working as a fitness advisor and the participants were operational firefighters. The interviews were held via Zoom (Gray et al. 2020) and recorded. Field notes were made during and after the interviews in this study. Two pilot interviews were conducted by LN with work colleagues within the fire service fitness department prior to the start of the interviews with the participants. Pilot interviews allowed LN to familiarise themselves with the questions and assess if any of the questions in the topic guide needed amending following feedback from colleagues. In addition, pilot interviews allowed for testing the run time of each interview and testing of the recording function to test the sound quality from both the researcher and the individual interviewed.

### Participants

All current operational firefighters for Essex county fire and rescue service who had previously been injured and returned to work were identified from records and invited to participate (*n* = 20). Records extended to the past 24 months. Twenty participants were emailed an invitation by LN to take part in an interview, along with the participant information sheet. Interested participants had an opportunity to ask questions via email or telephone prior to organising an interview date and time at a mutually convenient time. Prior to commencing the interview, the participant had a further opportunity to ask any questions prior to providing written consent via email. Consent was also audio recorded. Data saturation was determined when all pre-determined themes had been represented adequately in the data collected. (Saunders et al. 2018; Strauss and Corbin 1998).

### Data management

Data from all sources were maintained and stored on a password-protected laptop computer. All data were anonymised at source and no identifiable data was kept.

### Data analysis

The recordings were transcribed verbatim and then coded using NVIVO 12 software by LN (Richards 1999). The coding was checked and verified by AM. Thematic analysis of the data collected was undertaken using The Framework Method. The Framework Method has been developed specifically for applied research in which the objectives of the investigation are set a priori (Pope et al. 2000). The Framework Method allows for a systematic approach to qualitative analysis which provided the ability to compare and contrast data by themes across individual cases (Gale et al. 2013). The Framework Method consists of seven steps of data analysis (Table 1). LN sent the results framework to all participants to give them an overview of the results for interpretation.

**Table 1:** Use of The Framework Method during analysis of data

Step of analysis	Description
1. Transcription	The recordings of the interviews were transcribed verbatim by the chief investigator (LN)
2. Familiarisation with the interview	All recordings where relistened to and quality checked with the transcripts by LN
3. Coding	All transcripts were read line by line and codes were applied to the parts of the interviews that were deemed to be relevant by LN. The parts were coded in relation to the pre-existing themes which were informed by the topic guide. Open coding was also used during this process for parts of the interviews which were interesting but did not fit with the initial coding framework. This was to ensure that potential important pieces of data were not missed. Coding was reviewed and verified by AM (Fig. 1)
4. Developing a working analytical framework	Once all coding was completed, LN analysed the coding to establish that there were no new themes to add relevant to the research aims
5. Applying the analytical framework	The transcripts were then indexed, and codes were used relating to the pre-existing themes by LN. NVIVO 12 software was used to code the transcripts
6. Charting data into the framework matrix	The coded data from the transcripts was inputted into a final report, the quotations from the participants were numbered to keep anonymity. LN was assured that data saturation, in relation to the research aims, had been achieved and no new themes had been found from the final interviews
7. Interpreting the data	LN interpreted the coded data and explored the relationship between the pre-existing themes in relation to the research aims. From these, nine subthemes were identified

## Results

Twenty firefighters met the inclusion criteria and were invited to participate in the study. Of these, 12 (60%) agreed to participate (Table 2). No response was received from the remaining eight firefighters (40%) invited. Interviews lasted up to 30 min.

**Table 2:** Participants characteristics

Participant	Gender	Rank	Duty type	Type of injury	Time out of operational duties
1	Male	Firefighter	On-Call	Rotator cuff sprain	3 months
2	Female	Firefighter	Wholetime	Anterior cruciate ligament surgery	14 months
3	Male	Firefighter	On-Call	Neck and back sprain	5 months
4	Female	Firefighter	Wholetime	Broken wrist	3 months
5	Male	Crew Manager	Wholetime	Back sprain	2 months
6	Male	Firefighter	Wholetime	Knee surgery	3 months
7	Male	Firefighter	Wholetime	Knee sprain	1 month
8	Male	Firefighter	On-Call	Shoulder surgery	2 months
9	Male	Firefighter	Wholetime	Fractured wrist	2 months
10	Male	Firefighter	On-Call	Back sprain	3 months
11	Male	Firefighter	On-Call	Fractured thumb	6 months
12	Male	Watch Manager	Wholetime	Heart surgery	12 months

### Findings

**Fig. 1:**
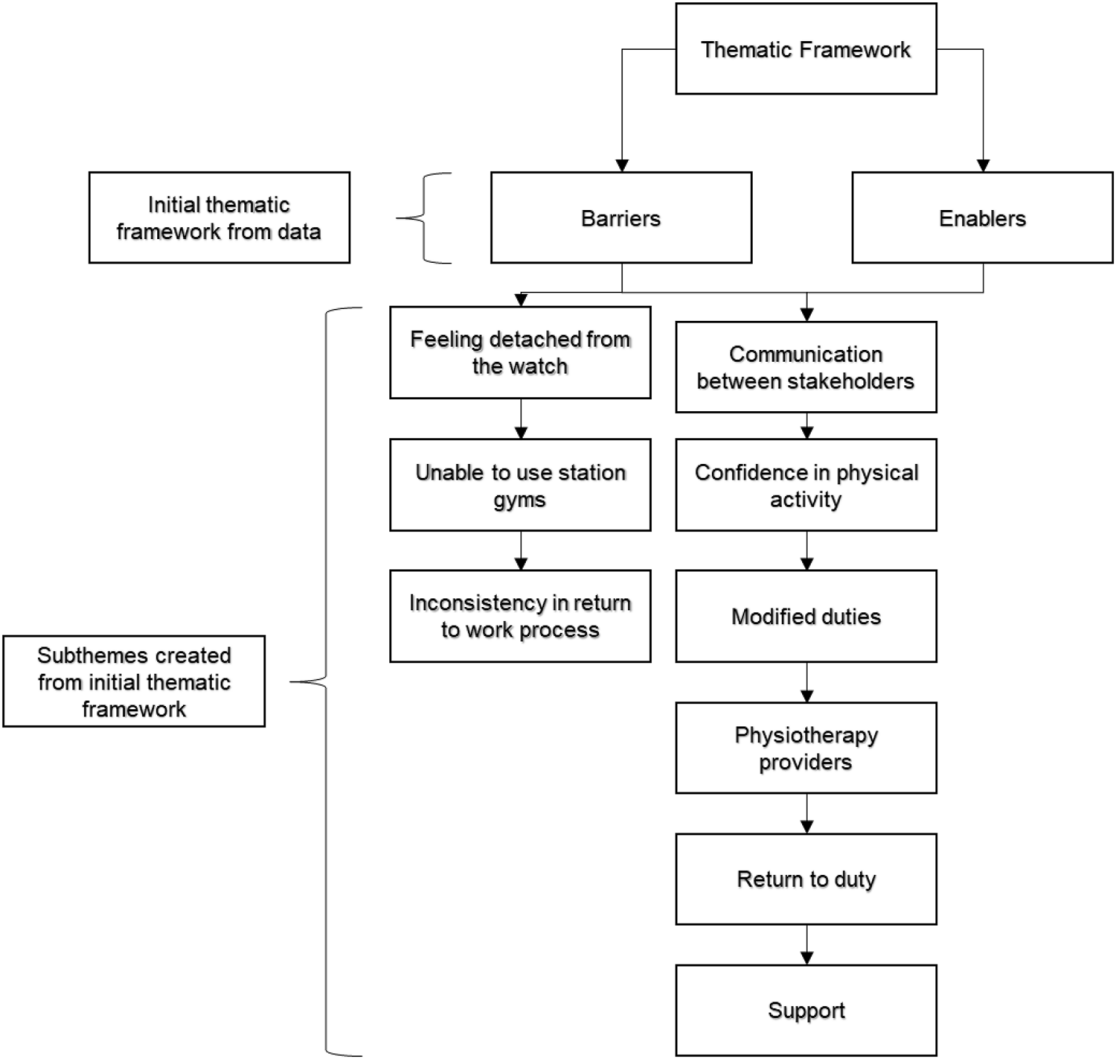
Mapping of the thematic framework

Two main themes were sought after transcription: barriers and facilitators. From these, nine subthemes were identified; (1) communication, (2) confidence in physical activity participation, (3) modified duties, (4) physiotherapy, (5) return to operational duties, (6) support, (7) inconsistency, (8) use of station gyms, (9) detachment from the watch (Fig. 1).

### Barriers

#### Theme one: communication

Communication between different departments involved in the return to work process was perceived as being a barrier:

“It could have helped with a quicker return if everyone were in communication with each other. I felt all different departments were separate and the lack of communication dragged the process along”.—Participant 3.

#### Theme two: confidence in physical activity participation

A common theme reported was a confidence to participate in physical activity following an injury was low.

“I started to take myself out for short jogs, but was nervous as hell doing it”—Participant 2.

“My confidence was completely shot if I’m honest. I was so worried about doing any damage that I did the bare minimum, which was frustrating because I kept comparing to how I was. Even though I wanted to get back to my original fitness, I just didn’t have the confidence to push myself.”—Participant 12.

#### Theme three: modified duties

Whilst recovering from their injury, some firefighters were given the opportunity to work on modified duties. However, other firefighters were not given this opportunity and because of this they perceived it as a barrier during their return to work experience.

“I would have loved to be able to return to work in a format where I could do some things and not others, that way I could still help out. Instead of this all or nothing approach.”—Participant 11.

#### Theme four: physiotherapy

All of those interviewed had some form of treatment from a physiotherapist during their rehabilitation. Some found that the expectations from the physiotherapists for recovery were not meeting work demands.

“The physio’s were mainly looking for weight-bearing movements and walking but I knew in the back of my mind what I would be required to do when returning to operational duties.”—Participant 5.

“They helped and I did benefit from them, however, I knew that the level I needed to reach was beyond their expected level from me”—Participant 6.

#### Theme five: return to operational duties

Once they had returned to operational duties, some firefighters felt that the aftercare from human resources (HR) could have been better.

“I felt like I was expected to just return to normal as if nothing had happened. I didn’t mind it, but it would have been nice for someone from HR to check in to see how I was doing.”—Participant 6.

Many firefighters reported that there needed to be an improvement in the aftercare following a return to work from injury.

“It would be good for the fitness team to create a training package where firefighters could go to and select a workout suitable for the equipment they have or body part they want to train. It could go up on the wall to make it easily accessible.” Participant 3.

#### Theme six: support

The support from the fire service varied across the firefighters interviewed. Some firefighters felt mistreated and that the service was putting barriers in their way to return to work.

“My manager was also fully aware that I needed to do a functional assessment so I guess it would have been nice for him to let me know to reduce the delay. If I had known I would have got it booked in advance for the day my sick certificate ran out. I just wanted to get back and it felt like there were hurdles put in my way for what was in my opinion a simple injury.”—Participant 7.

#### Theme seven: inconsistency

A common barrier reported was the inconsistency of the process for a firefighter to return to operational duties following an injury.

“I feel that there needs to be consistency in the service for return to work. So, no matter where you are based you are aware of what needs to be achieved to return to work. That way it would stop managers adding in extra assessments here and there because they feel like it.”—Participant 4.

Other firefighters reported that the return to work process needed to be clearer to increase consistency.

“I think there should be a clear guidance of if you’re off work for an injury you are required to do a return to work assessment with the fitness team. Because it would clear any confusion I experienced and also possibly reduce the amount of time of spent on modified duties.”—Participant 7.

#### Theme eight: use of station gyms

Whilst injured, many firefighters were not allowed on the station. This meant that they were unable to use the gym facilities during their recovery, which many perceived as a barrier.

“I didn’t have any weights at home to help increase my strength in my wrist which was a bit frustrating. It would have been nice to be able to go to the station to use the gym to help with my recovery or have the opportunity to have supervised gym sessions with someone from the fitness team maybe?”—Participant 9.

#### Theme nine: detachment from the watch

Being away from the station also meant that injured firefighters were unable to meet up with the colleagues on their watch. This was reported as a barrier by many.

“I wasn’t allowed on station. I was considered a visitor and lost contact with the watch, the meals together, the environment, the banter. I felt completely disconnected with the watch. Normally, you are there to see the morning tests and routines but being away I feel separated. We have WhatsApp but it’s not the same as face to face contact.”—Participant 2.

“It was frustrating being off that long, not being able to see my friends down at the station, I felt a bit like I was being punished for being injured. I felt really detached from the station.”—Participant 10.

### Facilitators

#### Theme one: communication

Interviews found that communication regarding the return to work process and requirements to pass the functional assessment was good between different stakeholders including line managers and occupational health. This was a facilitator with their return to work process.

“I spoke to occupational health about what I was required to do to return to work and they said it would be a functional assessment, that’s when I contacted you and asked what was involved. From there I worked with the physio to build up my fitness levels, specifically in my shoulder.”—Participant 8.

#### Theme two: confidence in physical activity participation

For some firefighters, their confidence was affected but they were comfortable participating in physical activity, building their strength back up gradually.

“Going back to running I was very cautious, so I started with a light jog and increased the speed slightly each week. Confidence to train on my own was okay, it was just having the confidence to push my knee.”—Participant 6.

#### Theme three: modified duties

Whilst recovering from their injury, some firefighters were given the opportunity to work on modified duties. This was perceived as a facilitator during their return to work experience.

“I was allowed back into the training department to do light duties, this involved admin, cleaning equipment, nothing too strenuous but got me back in the rhythm of working again. I also was allowed to work flexible times as my medication made me tired towards the latter part of the afternoon.”—Participant 12.

#### Theme four: physiotherapy

Some firefighters used private physiotherapy providers who had a contract with the fire service to allow six free treatment sessions for each firefighter per injury. These were perceived as a facilitator for many firefighters.

“For me, the physio didn’t just help with the physical side but also the mental side for reassurance my injury was getting better.”—Participant 3.

“They were very good in my opinion, they assessed my shoulder and we worked towards strengthening it for the functional assessment.”—Participant 8.

#### Theme six: support

Many firefighters reported that they felt supported throughout their time off being injured and during their return to work process.

“In terms of getting me back on the run, I was supported from my line manager, the service, the fitness team and the occupational health team. With sufficient time to get back onto the run and come along to do a return to work assessment.”—Participant 5.

## Discussion

The aim of this study was to explore the psychosocial barriers and facilitators during the return to work process following an injury for a firefighter. Nine sub-themes were identified; communication, confidence in physical activity participation, modified duties, physiotherapy, return to operational duties, support, inconsistency in the return to work process, use of station gyms and detachment from the watch.

The findings suggest that providing station access to see their colleagues could increase social contact whilst being off sick. The reported feelings of detachment and frustration from being away from the fire station and their colleagues in this study are similar to those experienced in other active populations including athletes (Crossman 1997; Barber-Westin et al. 2019). Providing access to see colleagues could help to decrease the feelings of detachment from the watch. Examples could include joining meals or attending educational training lectures where no physical activity is required.

Future practice should consider allowing injured firefighters access to gym facilities on their fire stations to aid with their rehabilitation. An individual’s muscular strength and aerobic fitness levels can decrease with physical inactivity (Prang et al. 2015) and the majority of fire services in the United Kingdom require their firefighters to achieve a maximal aerobic capacity level (*V*O_2 max_) of 42.3 ml/kg/min as a minimum to be considered safe to carry out operational duties (Siddall et al. 2016). A strength standard of a 32 kg shoulder press and a 60 kg rope pull down has also been recommend (Stevenson et al. 2017). Sport scientists and physiotherapists need to consider the basic physical requirements an operational firefighter needs to achieve before returning to duty in addition to injury rehabilitation. Therefore, restricting access to gym facilities could be a barrier to achieving these standards for returning to operational duties, especially as resistance training has been identified as critical for the recovery of musculoskeletal function following injury in athletic populations (Wayda et al. 1998).

However, providing access to station gym facilities could be further enhanced with a training plan. At present, injured firefighters are not given a fitness training plan to help with their return to work preparation unless they specifically request one from a qualified professional, in this case a fitness advisor, to help increase the effectiveness of the firefighter’s injury rehabilitation (Andersen et al. 2019). The multidisciplinary team, including physiotherapists, occupational health, the fitness team and line management, should keep in regular contact with the firefighter monitor the firefighter’s progression through the exercise programme and progress as required.

To improve the development of an exercise plan for firefighters, good communication between physiotherapists and the fire service occupational health department is needed (Andersen et al. 2019). Communication was a barrier reported in this study, specifically between physiotherapists, occupational health, fitness advisors and managers. Firefighters all had treatment from a physiotherapist before they were referred to the ‘in house’ occupational health service and fitness team to carry out a functional assessment. Once they were referred, firefighters were responsible to update occupational health on their progress. Leaving firefighters to be solely responsible to provide this progress update could result in important information being missed. Instead, if the physiotherapist liaised directly with occupational health and the fitness team a professional update could be provided to ensure all information is handed over. This improved communication could also help improve physiotherapists’ awareness of the physical expectations required of a firefighter during their return to work assessment and align rehabilitation goals with strength and aerobic goals. This could help give the injured firefighter a sense of control and increased motivation as they could monitor their strength and aerobic fitness levels (Wayda et al. 1998).

Motivation can also come from the support of management providing a positive experience for individuals returning to work following an injury (Andersen et al. 2019). Our findings showed an inconsistency in management support across the fire service; some managers in this study were perceived as enablers for firefighters to return to work, others were perceived as barriers. Inconsistency between managers was evident. Some offered firefighters the opportunity to perform modified duties; others were not. This could relate to the duty system; whole-time firefighters work full time for the fire service, on-call firefighter’s work part time on a pager and are employed elsewhere. Providing whole-time firefighters modified duties could be easier as they do not have alternate employment. Future practice should allow all firefighters to be given the opportunity where possible to perform modified duties. This could include carrying out safety checks and station administration tasks regardless of their duty system. This would increase a firefighter’s interaction with their colleagues and manager and prevent feelings of isolation.

Consistency would be increased by the introduction of a guidance framework for a return to work following injury. For example, the creation of a flow chart staging each process of a return from injury, who is responsible at that stage and what their role is during that process (Slevin and Roberts 1987). This would also help communication expectations between physiotherapists, occupational health, fitness advisors, managers and firefighters. This would help ensure all firefighters received the same level of support whilst recovering from an injury.

### Strengths and limitations

This study included current operational firefighters from the United Kingdom. There was representation from both whole-time and on-call duty systems. All interviews were conducted via video call without the need for travel or expenditure. The study only used one fire service. It is not known if such barriers and facilitators are the same across the fire service.

The use pre-determined themes during the semi-structured interviews could have prevented any other themes from emerging from the firefighters return to work experience which could have resulted in them being missed during the analysis.

## Conclusion

This study provided the perceived barriers and facilitators firefighters faced during their return to work process following an injury. Consideration should be made for the consistency of procedures followed during an individual’s return to work following an injury. This could include communication between the occupational health department, the fitness team and the physiotherapists to provide a rehabilitation plan for the firefighter. Access to the fire station should also be considered to encourage social contact and allow physical training as part of their rehabilitation in preparation for the functional assessment. Further research is needed to understand if the themes identified in this study are the same for other fire services. Further research is also needed to understand how the findings may be best implemented within the fire service.

## Supplementary Information

Below is the link to the electronic supplementary material.

Supplementary file1 (DOCX 13 kb)

## Data Availability

All data generated or analysed during this study are included in this published article [and its supplementary information files].
