# Supplementary material for: Psychosocial barriers and facilitators for a successful return to work following injury within firefighters
Source: Int Arch Occup Environ Health. 2021 May 11;95(2):331–9. doi: 10.1007/s00420-021-01712-z (PMC8795041; doi:10.1007/s00420-021-01712-z)
Supplement: Supplementary file 1 — Supplementary file1 (DOCX 13 kb) [file 420_2021_1712_MOESM1_ESM.docx]

**TOPIC GUIDE FOR SEMI-STRUCTURED INTERVIEWS**

1. **Introduction**

- Introduction to researcher and study topic
- Explanation of the aim of the study
- Explain confidentiality and anonymity
- Explain recording length (up to 30 minutes) and nature of discussion
- Go through consent issues and explain they may withdraw at any time and they do not have to answer any interviews they would prefer not to
- Check whether they have any questions
- Check they are happy to continue

1. **Experience of returning to work following an injury**

- Describe overall experience
- Establish any perceived barriers faced during their experience
- Establish any perceived enablers faced during their experience
- Where they feel their confidence is to participate in physical activity alone.
- Where they feel areas for improvement lie during the return to work process – Fitness, Occupational health, HR, management support, physio provisions.
- Check for any unintended consequences
- Check for any other comments

1. **In conclusion**

- Summarise and check key issues
- Thank the participant for their time.
- Reiterate confidentiality

**END RECORDING**
